# IGFBP2 attenuates high glucose-induced endothelial dysfunction through PI3K/Akt/eNOS pathway activation

**DOI:** 10.1042/BSR20260182

**Published:** 2026-10-29

**Authors:** Xiaoyu Song, Wentao Zhong, Chengqian Chen, Hao Zheng, Zhuo Li

**Affiliations:** 1 Department of Endocrinology and Metabolism, The First Hospital of Jilin University, Changchun, China; 2 Department of Hematology, The First Hospital of Jilin University, Changchun, China

**Keywords:** endothelial dysfunction, eNOS, HUVECs, IGFBP2, PI3K/Akt signaling

## Abstract

Endothelial dysfunction induced by hyperglycemia is a key event in diabetic vascular complications and is closely associated with impaired PI3K/Akt/endothelial nitric oxide synthase (eNOS) signaling. Insulin-like growth factor-binding protein 2 (IGFBP2) has been implicated in metabolic regulation and cell survival, but its role in high glucose (HG)-induced endothelial injury remains unclear. In the present study, human umbilical vein endothelial cells (HUVECs) were exposed to HG (33 mM) for 24 h and then treated with recombinant human IGFBP2 (rhIGFBP2, 200 ng/ml) for 60 min. HG reduced Akt/eNOS phosphorylation and nitric oxide (NO) production, increased the expression of ICAM-1, VCAM-1, and SELE, promoted interleukin-6, interleukin-8, monocyte chemoattractant protein-1 secretion, and enhanced apoptosis. rhIGFBP2 attenuated these HG-induced changes by restoring PI3K/Akt/eNOS signaling and NO production, reducing inflammatory activation, and decreasing apoptotic cell percentage. The effect of rhIGFBP2 on Akt/eNOS phosphorylation and NO production was broadly comparable to that of insulin under HG conditions. HG also increased p38 MAPK phosphorylation, whereas rhIGFBP2 showed only a nonsignificant decreasing trend. Importantly, PI3K inhibition with LY294002 abolished rhIGFBP2-induced Akt/eNOS activation, supporting the involvement of the PI3K/Akt/eNOS pathway. These findings suggest that IGFBP2 protects against HG-induced endothelial dysfunction mainly by activating PI3K/Akt/eNOS signaling and may represent a potential mediator of endothelial protection under diabetic conditions.

## Introduction

Diabetic vascular complications are the leading cause of disability and mortality in diabetic patients and are among the earliest and most prevalent manifestations of the disease [1,2]. The pathological basis of these complications is endothelial dysfunction induced by persistent hyperglycemia. Disruption of glucose homeostasis can trigger endothelial dysfunction [3,4], which is characterized by reduced nitric oxide (NO) bioavailability and impaired vasodilation [5–7]. This condition is accompanied by a series of pathological alterations, including dysregulated angiogenesis [8,9], exacerbated inflammation, increased vascular permeability, and enhanced susceptibility to low-density lipoprotein oxidation [10–12]. Therefore, identifying novel therapeutic targets to protect endothelial cells and improve their function is of significant scientific and clinical value for preventing and treating diabetic vascular diseases.

The PI3K/Akt/endothelial nitric oxide synthase (eNOS) signaling pathway is a central pathway for maintaining endothelial cell homeostasis [13,14]. Akt activation phosphorylates eNOS, promoting the production of NO, a molecule with vasodilatory, anti-inflammatory, and anti-apoptotic properties [15–17]. Hyperglycemia induces excessive generation of intracellular reactive oxygen species (ROS), which exacerbates endothelial dysfunction by impairing the antioxidant system and promoting inflammatory responses [18]. Notably, the PI3K/Akt/eNOS pathway also regulates ROS-associated endothelial injury [15,19]. Substantial evidence indicates that this pathway is suppressed under high glucose (HG) conditions, representing a key event in the development of endothelial dysfunction [20–22]. Consequently, discovering endogenous protective factors that can activate this pathway is crucial for preserving endothelial cell function.

Insulin-like growth factor-binding protein 2 (IGFBP2), a key member of the IGF system, has traditionally been viewed as a regulator of IGF bioavailability. However, recent studies have revealed that IGFBP2 possesses various IGF-independent biological functions, including the regulation of cell metabolism, growth, and survival [23–25]. Research in fields such as oncology suggests that IGFBP2 can activate the PI3K/Akt signaling pathway [26–28]. However, within the cardiovascular system, while some studies link IGFBP2 to cardiovascular disease risk [29,30], its specific role and underlying mechanisms in diabetic vascular endothelial cell injury remain unclear. It is unknown whether IGFBP2 can exert a direct protective effect by modulating the Akt/eNOS pathway.

In summary, we hypothesized that IGFBP2 protects human umbilical vein endothelial cells (HUVECs) from HG-induced injury by activating the PI3K/Akt/eNOS signaling pathway. Using an *in vitro* HG stimulation model, the present study evaluated the effects of recombinant human IGFBP2 (rhIGFBP2) on HUVEC viability, inflammatory response, and key signaling pathways. The dependence on the pathway was further verified using the PI3K-specific inhibitor LY294002. Our study is the first to demonstrate that IGFBP2 directly protects against endothelial dysfunction under diabetic conditions and to elucidate the underlying molecular mechanism. These findings identify IGFBP2 as a novel potential target for preventing and treating diabetic vascular complications.

## Materials and methods

### Materials

rhIGFBP2 protein was purchased from R&D Systems (Minneapolis, MN, U.S.A.). Recombinant human insulin was purchased from Procell Life Science & Technology Co., Ltd. (Wuhan, Hubei, China) and used at a final concentration of 1 μg/ml. The PI3K inhibitor LY294002 (#S1105) was obtained from Selleck Chemicals (Houston, TX, U.S.A.). LY294002 was dissolved in DMSO to prepare a stock solution, and rhIGFBP2 was dissolved in PBS to prepare a stock solution. The final concentration of the vehicle was kept consistent across the corresponding experimental groups. The HG medium was prepared using 33 mM D-glucose, whereas the normal glucose (NG) control medium contained 5.5 mM D-glucose. Mannitol was used at 27.5 mM to prepare the osmotic control (OSM) medium.

Antibodies against phospho-Akt (#4060), pan-Akt (#4691), PI3 kinase p85 (#4257), phospho-p38 MAPK, total p38 MAPK, α-tubulin, anti-mouse IgG HRP-linked secondary antibody (#7076), and anti-rabbit IgG HRP-linked secondary antibody (#7074) were purchased from Cell Signaling Technology (Danvers, MA, U.S.A.). Antibodies against phospho-eNOS (#AP0421), total eNOS (#A2098), and phospho-PI3K p85 (Tyr458)/p55 (Tyr199) (#AP0427) were purchased from ABclonal Technology Co., Ltd. (Wuhan, Hubei, China). GAPDH (#60004-1-Ig), human IL-6 ELISA kit, human IL-8 ELISA kit, and human MCP-1 ELISA kit were purchased from Proteintech Group, Inc. (Wuhan, Hubei, China). The Annexin V-FITC/PI apoptosis detection kit was purchased from Elabscience Biotechnology Co., Ltd. (Wuhan, Hubei, China). The RNA extraction kit (#LS1040), RT Master Mix (#LS2050), and qPCR Master Mix (#LS2062) were from Promega (Madison, WI, U.S.A.). The BCA Protein Assay Kit (#P0012S) was from Beyotime Biotechnology.

### Cell culture

The HUVECs (#CL-0675) and specialized HUVEC medium (#CM-0675) were purchased from Procell Life Science & Technology Co., Ltd (Wuhan, Hubei, China). The cell line was tested negative for mycoplasma contamination by the supplier using the PCR-based method. Cell line authentication was performed by the vendor using short tandem repeat profiling. All experiments were performed using cells between passages 3 and 8. Cells were cultured at 37°C in a humidified incubator with 5% CO_2_. When cells reached 80%–90% confluence, they were passaged using 0.25% trypsin-EDTA.

THP-1 human monocytic cells were purchased from Procell Life Science & Technology Co., Ltd. (Wuhan, Hubei, China) and cultured in RPMI-1640 medium supplemented with 10% fetal bovine serum and 1% penicillin–streptomycin. Cells were maintained in suspension at 37°C in a humidified incubator with 5% CO_2_ and passaged every 4–5 days to maintain logarithmic growth. THP-1 cells in the logarithmic growth phase were used for the monocyte adhesion assay.

### Experimental grouping and treatments

#### Concentration- and time-effect experiments

To determine the optimal intervention conditions for rhIGFBP2, HUVECs were treated with different concentrations of rhIGFBP2 (0, 10, 50, 100, 200, and 500 ng/ml) for 1 h. After 200 ng/ml was selected as the lowest effective concentration, cells were treated with 200 ng/ml rhIGFBP2 for different durations (0, 15, 30, 60, and 120 min). The protein expression level of p-Akt was detected by Western blot analysis.

#### High glucose model validation experiment

Cells were divided into three groups: NG group, OSM group, and HG group. After 24 h of treatment, proteins were extracted, and the inhibitory effect of HG on Akt phosphorylation was verified by Western blot analysis.

#### Validation of the protective effect of rhIGFBP2

Cells were divided into five groups: NG, OSM, HG, HG + vehicle, and HG + rhIGFBP2. After 24 h of HG stimulation, rhIGFBP2 (200 ng/ml) or vehicle was added for 60 min. Cell supernatants, RNA, and proteins were collected for subsequent analysis.

#### Insulin comparison experiment

To compare the effects of insulin and rhIGFBP2 on the Akt/eNOS/NO pathway under HG conditions, cells were divided into four groups: NG, HG, HG + insulin, and HG + rhIGFBP2. After 24 h of HG stimulation, cells were treated with insulin (1 μg/ml) or rhIGFBP2 (200 ng/ml) for 60 min. The phosphorylation levels of Akt and eNOS were detected by Western blot analysis, and NO production was measured in the cell culture supernatant.

#### Inflammatory marker experiment

To further evaluate endothelial inflammatory activation, cells were divided into NG, OSM, HG, and HG + rhIGFBP2 groups. After treatment, cell culture supernatants were collected for interleukin-6(IL-6), interleukin-8 (IL-8), and monocyte chemoattractant protein-1 (MCP-1) measurement by ELISA, and total RNA was extracted for analysis of SELE mRNA expression by qPCR.

#### Apoptosis analysis experiment

To assess apoptosis under HG conditions, cells were divided into NG, HG, and HG + rhIGFBP2 groups. After treatment, apoptosis was evaluated using Annexin V-FITC/PI staining followed by flow cytometry.

#### p38 MAPK phosphorylation experiment

To examine whether p38 MAPK was involved in the response to HG and rhIGFBP2 treatment, cells were divided into NG, HG, and HG + rhIGFBP2 groups. The phosphorylation level of p38 MAPK was detected by Western blot analysis.

#### Pathway inhibitor validation experiment

To verify pathway dependency, the PI3K inhibitor LY294002 was used. Cells were divided into six groups: NG + vehicle, HG + vehicle, HG + rhIGFBP2 + vehicle, NG + LY294002, HG + LY294002, and HG + rhIGFBP2 + LY294002. LY294002 (20 μM) or vehicle was added 30 min before rhIGFBP2 treatment.

### Cell viability assay

Cell viability was assessed using a Cell Counting Kit-8 (CCK-8). Cells were seeded in 96-well plates at a density of 5 × 10^3^ cells per well. After treatments according to the aforementioned groupings, 10 μl of CCK-8 solution was added to each well, followed by incubation for 2 h in the culture incubator. The absorbance of each well was measured at a wavelength of 450 nm using a microplate reader.

### Nitric oxide measurement

NO production in HUVECs was measured using a commercial NO assay kit based on the Griess reaction (Beyotime, China). After treatment, cell culture supernatants were collected and centrifuged at 12,000×***g*** for 10 min to remove debris. Nitrate was converted to nitrite according to the manufacturer’s instructions. Absorbance was measured at 540 nm using a microplate reader. NO levels were calculated from a sodium nitrite standard curve and normalized to total protein concentration.

### Monocyte adhesion assay

A monocyte adhesion assay was performed to assess endothelial inflammatory activation. HUVECs were seeded on glass coverslips and cultured until they reached approximately 90% confluence. After the indicated treatments, THP-1 cells were labeled with 5 μM calcein-AM at 37°C for 30 min in the dark. The labeled THP-1 cells were washed with PBS and then added to HUVEC monolayers at a density of 1 × 10^5^ cells/well. After incubation for 60 min at 37°C, non-adherent THP-1 cells were gently removed by washing three times with PBS. The cells were fixed with 4% paraformaldehyde, and HUVECs were counterstained with DAPI and CoralLite-labeled phalloidin to visualize nuclei and the cytoskeleton. Images were acquired using a fluorescence microscope under identical exposure settings.

### Quantitative real-time PCR

Total RNA was extracted from cells using the RNA extraction kit according to the manufacturer’s instructions. RNA concentration and purity were determined using a NanoDrop spectrophotometer. One microgram of total RNA was reverse-transcribed into cDNA using the RT Master Mix for qPCR. Amplification was performed using the qPCR Master Mix on a QuantStudio 5 Real-Time PCR System. The PCR cycling conditions were as follows: initial denaturation at 95°C for 30 s, followed by 40 cycles of 95°C for 10 s and 60°C for 30 s. The relative mRNA expression levels of VCAM-1, ICAM-1, and SELE were calculated using the 2^−ΔΔCT^ method, with β-actin as the internal control.

The primers were synthesized by Sangon Biotech (Songjiang, Shanghai, China), and their sequences are as follows:

VCAM-1: Forward, 5′-CACTGAATGGGAAGGTGACG-3′; Reverse, 5′-CAGTAAGTCTATCTCCAGCC-3′.

ICAM-1: Forward, 5′-GGTGTATGAACTGAGCAAT-3′; Reverse, 5′-CCTGGCAGCGTAGGGTAAG-3′.

SELE: Forward, 5′-CTGCAATGTGGTTGAGTGTG-3′; Reverse, 5′-GTACAGGTTGTGTTCCATGG-3′.

β-actin: Forward, 5′-GCACCCAGCACAATGAAGA-3′; Reverse, 5′-TGGAGGGGCCGGACTCGTC-3′.

### Measurement of inflammatory cytokines

The concentrations of IL-6, IL-8, and MCP-1 in cell culture supernatants were measured using commercial ELISA kits according to the manufacturer’s instructions. Briefly, supernatants were collected after treatment and centrifuged to remove cell debris. Standards and samples were added to ELISA plates, and absorbance was measured at 450 nm using a microplate reader. Cytokine concentrations were calculated from standard curves and expressed as pg/ml.

### Flow cytometry analysis of apoptosis

Cell apoptosis was detected using an Annexin V-FITC/PI apoptosis detection kit according to the manufacturer's instructions. After the indicated treatments, both floating and adherent cells were collected. Adherent cells were harvested using EDTA-free trypsin, washed twice with cold PBS, and resuspended in binding buffer. Cells were then stained with Annexin V-FITC and propidium iodide for 15 min at room temperature in the dark. Samples were analyzed by flow cytometry within 1 h after staining. Early apoptotic cells were defined as Annexin V-positive and PI-negative cells, and late apoptotic cells were defined as Annexin V-positive and PI-positive cells. The percentage of total apoptotic cells was calculated as the sum of early and late apoptotic cell percentages.

### Western blot analysis

Total protein was extracted from cells using RIPA lysis buffer supplemented with protease and phosphatase inhibitor cocktails. The protein concentration was determined using the BCA method. Equal amounts of protein samples were separated by SDS-PAGE and transferred to PVDF membranes. The membranes were blocked with Rapid Blocking Buffer for 10 min at room temperature and then incubated with the corresponding primary antibodies at 4°C overnight. After washing with TBST, the membranes were incubated with HRP-conjugated secondary antibodies at room temperature for 1 h. The blots were visualized using a chemiluminescent substrate and imaged. Band intensities were quantified using ImageJ software. Phosphorylation levels were calculated as the ratio of phosphorylated protein to the corresponding total protein, including p-PI3K/PI3K, p-Akt/Akt, p-eNOS/eNOS, and p-p38/p38. GAPDH or α-tubulin was used as the loading control.

### Statistical analysis

All experiments were independently repeated three times. Data are presented as the mean ± standard deviation (SD). Statistical analyses were performed using GraphPad Prism software (version 10.0; GraphPad Software, San Diego, CA, U.S.A.). Differences among multiple groups were analyzed using one-way ANOVA followed by Tukey’s post hoc test. A value of *P<*0.05 was considered statistically significant.

## Results

### rhIGFBP2 stimulates Akt phosphorylation in a dose- and time-dependent manner

To determine whether rhIGFBP2 activates Akt signaling in HUVECs and to establish the optimal intervention conditions, we first performed concentration- and time-response experiments. Treatment with increasing concentrations of rhIGFBP2 for 1 h enhanced Akt phosphorylation without markedly altering total Akt protein expression (Figure 1A). Quantitative analysis showed that the p-Akt/Akt ratio was significantly increased beginning at 200 ng/ml and reached the highest level at 500 ng/ml (Figure 1B). Therefore, 200 ng/ml was selected as the lowest effective concentration for subsequent experiments.

**Figure 1:**
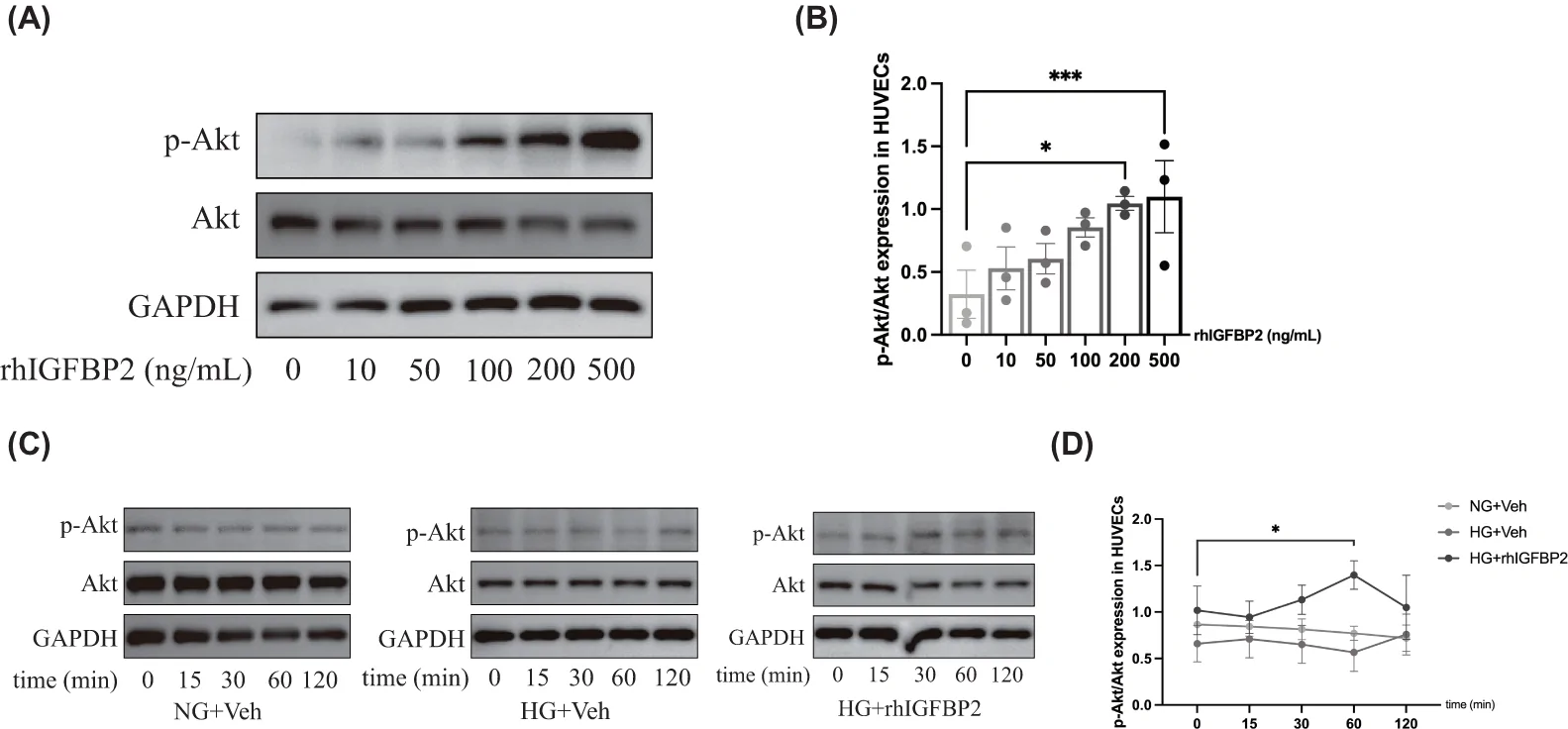
rhIGFBP2 stimulates Akt phosphorylation in HUVECs in a dose- and time-dependent manner (**A**) Representative Western blot images showing p-Akt, total Akt, and GAPDH protein levels in HUVECs treated with increasing concentrations of rhIGFBP2 (0, 10, 50, 100, 200, and 500 ng/ml) for 1 h. (**B**) Quantification of the p-Akt/Akt ratio in response to different concentrations of rhIGFBP2. (**C**) Representative Western blot images showing p-Akt, total Akt, and GAPDH protein levels in HUVECs under NG + Veh, HG + Veh, and HG + rhIGFBP2 conditions at the indicated time points. (**D**) Quantification of the p-Akt/Akt ratio during the time-course experiment. NG, normal glucose; HG, high glucose; Veh, PBS vehicle for rhIGFBP2; rhIGFBP2, recombinant human IGFBP2. Data are presented as the mean ± SD from three independent experiments. *P<0.05, ***P<0.001, as indicated.

We next examined the time course of Akt activation. Under HG conditions, rhIGFBP2 treatment at 200 ng/ml induced a time-dependent increase in Akt phosphorylation, with the most pronounced effect observed at 60 min (Figure 1C,D). In contrast, Akt phosphorylation remained relatively stable in the NG + vehicle and HG + vehicle groups during the same time course. Based on these results, rhIGFBP2 treatment at 200 ng/ml for 60 min was used in the following experiments.

### HG specifically suppresses Akt activation and induces endothelial dysfunction in HUVECs

To establish an *in vitro* model of hyperglycemia-induced endothelial dysfunction, HUVECs were exposed to NG, OSM, or HG conditions for 24 h. Compared with NG, HG markedly reduced Akt phosphorylation, whereas OSM did not significantly affect the p-Akt/Akt ratio (Figure 2A,B). These findings indicate that the inhibitory effect on Akt activation was mainly attributable to HG rather than hyperosmolarity.

**Figure 2:**
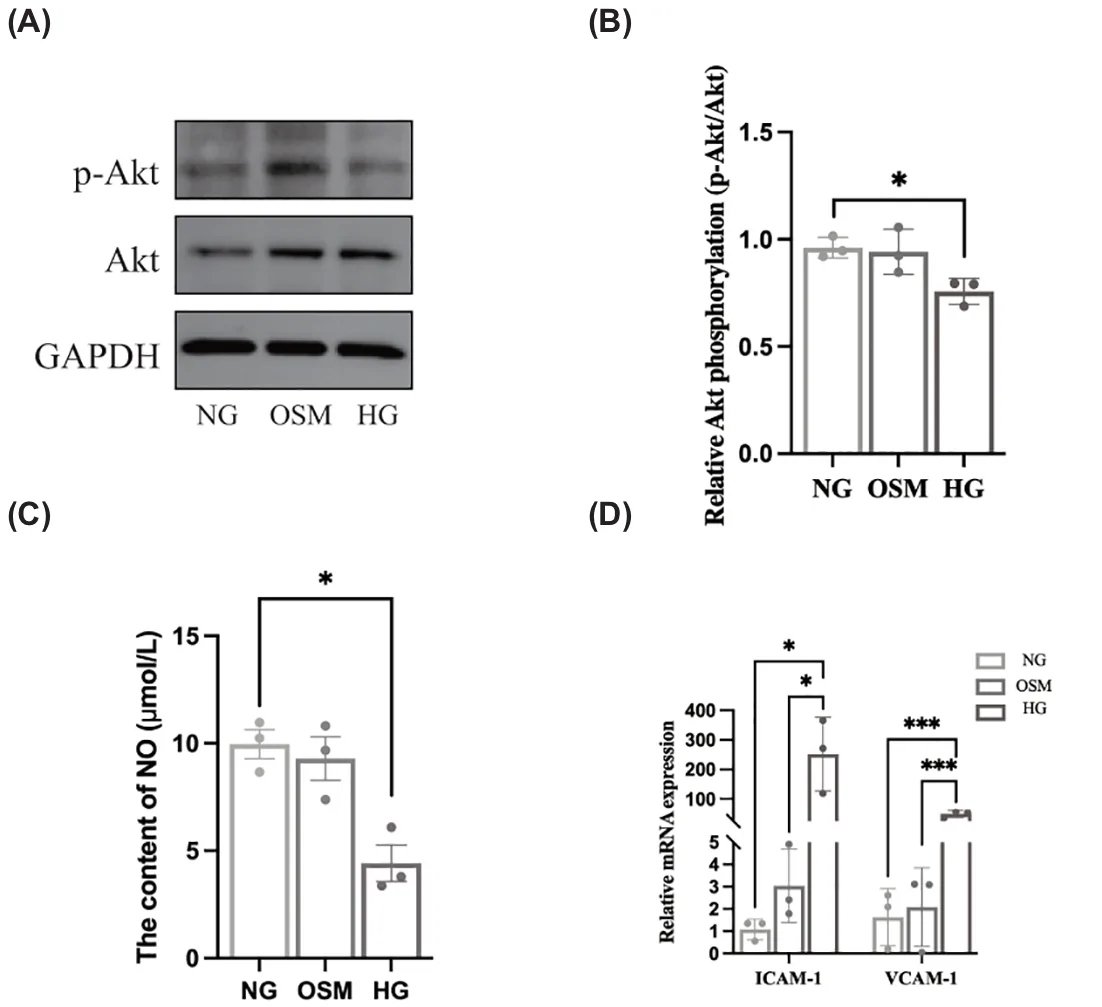
HG specifically suppresses Akt activation and induces endothelial dysfunction in HUVECs (**A**) Representative Western blot images showing p-Akt, total Akt, and GAPDH protein levels in HUVECs cultured under NG, OSM, or HG conditions for 24 h. (**B**) Quantification of the p-Akt/Akt ratio. (**C**) NO production in the cell culture supernatant. (**D**) Relative mRNA expression levels of ICAM-1 and VCAM-1 measured by qPCR. NG, normal glucose; OSM, osmotic control; HG, high glucose; NO, nitric oxide; ICAM-1, intercellular adhesion molecule-1; VCAM-1, vascular cell adhesion molecule-1. Data are presented as the mean ± SD from three independent experiments. *P<0.05, as indicated.

We further assessed endothelial functional impairment. HG significantly decreased NO production compared with NG, while OSM showed no significant effect (Figure 2C). In addition, HG markedly increased the mRNA expression of the adhesion molecules ICAM-1 and VCAM-1, whereas OSM did not induce a comparable inflammatory response (Figure 2D). These results confirmed that HG stimulation successfully induced endothelial dysfunction characterized by impaired Akt signaling, reduced NO production, and enhanced inflammatory activation.

### rhIGFBP2 reverses HG-induced endothelial dysfunction in HUVECs

We next evaluated endothelial inflammatory activation. HG and HG + vehicle markedly increased ICAM-1 and VCAM-1 mRNA expression, whereas OSM did not induce a comparable increase. rhIGFBP2 treatment attenuated the HG-induced up-regulation of these adhesion molecules (Figure 3A). In addition, HG markedly up-regulated SELE mRNA expression, while rhIGFBP2 reduced this increase (Figure 3B), further supporting the inhibitory effect of rhIGFBP2 on HG-induced endothelial inflammatory activation.

**Figure 3:**
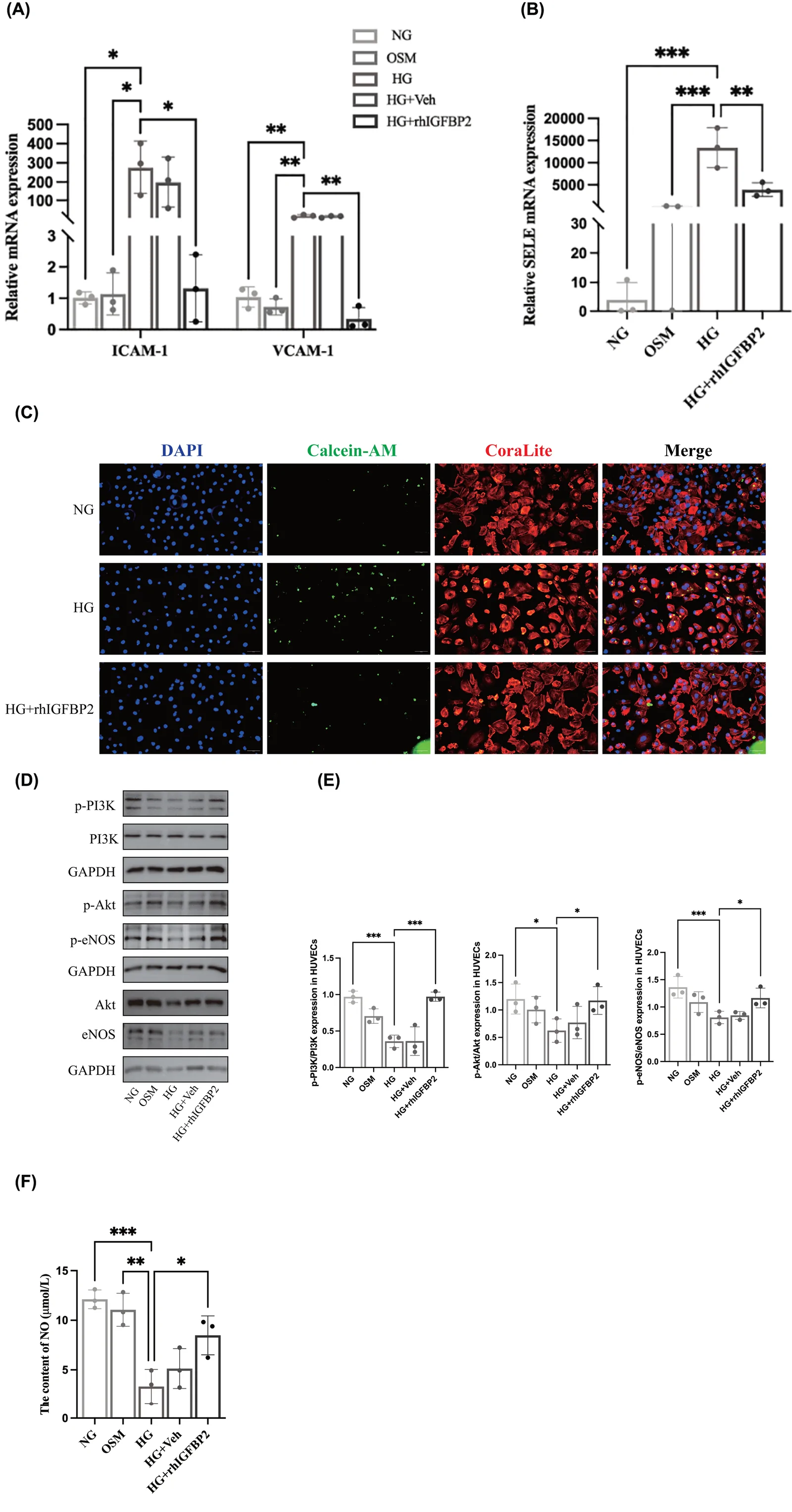
rhIGFBP2 attenuates HG-induced endothelial inflammatory activation and restores PI3K/Akt/eNOS signaling in HUVECs (**A**) Relative mRNA expression levels of ICAM-1 and VCAM-1 in HUVECs under NG, OSM, HG, HG + Veh, and HG + rhIGFBP2 conditions. (**B**) Relative mRNA expression level of SELE under NG, OSM, HG, and HG + rhIGFBP2 conditions. (**C**) Representative fluorescence images of monocyte adhesion to HUVECs. HUVEC nuclei were stained with DAPI (blue), THP-1 monocytes were labeled with Calcein-AM (green), and the HUVEC cytoskeleton was stained with CoralLite-labeled phalloidin (red). Scale bar = 100 μm. (**D**) Representative Western blot images showing p-PI3K, total PI3K, p-Akt, total Akt, p-eNOS, total eNOS, and GAPDH protein levels. (**E**) Quantification of the p-PI3K/PI3K, p-Akt/Akt, and p-eNOS/eNOS ratios. (**F**) NO production in the cell culture supernatant. NG, normal glucose; OSM, osmotic control; HG, high glucose; Veh, PBS vehicle for rhIGFBP2; rhIGFBP2, recombinant human IGFBP2; SELE, selectin E; NO, nitric oxide. Data in A, B, E, and F are presented as the mean ± SD from three independent experiments. Images in C are representative fluorescence images. *P<0.05, **P<0.01, ***P<0.001, as indicated.

Consistently, representative fluorescence images from the monocyte adhesion assay showed visibly increased adhesion of Calcein-AM-labeled THP-1 monocytes to HUVEC monolayers after HG exposure. Fewer adherent THP-1 cells were observed in the HG + rhIGFBP2 group compared with the HG group (Figure 3C), supporting the protective effect of rhIGFBP2 against HG-induced endothelial inflammatory activation.

To further investigate the underlying mechanism, we examined the PI3K/Akt/eNOS signaling pathway. HG suppressed the phosphorylation of PI3K, Akt, and eNOS, while rhIGFBP2 restored the phosphorylation of these signaling proteins under HG conditions (Figure 3D,E). Functionally, rhIGFBP2 also rescued the HG-induced decrease in NO production (Figure 3F). Together, these findings indicate that rhIGFBP2 protects against HG-induced endothelial dysfunction, at least in part, by restoring PI3K/Akt/eNOS signaling and NO production.

### rhIGFBP2 suppresses HG-induced inflammatory cytokine secretion

To further characterize the inflammatory response induced by HG, we measured inflammatory cytokines in the cell culture supernatant. HG significantly increased the secretion of IL-6, IL-8, and MCP-1 compared with NG and OSM conditions. rhIGFBP2 treatment significantly reduced the HG-induced elevation of these inflammatory cytokines (Figure 4A–C). These results provide additional evidence that rhIGFBP2 suppresses HG-induced inflammatory activation in HUVECs.

**Figure 4:**
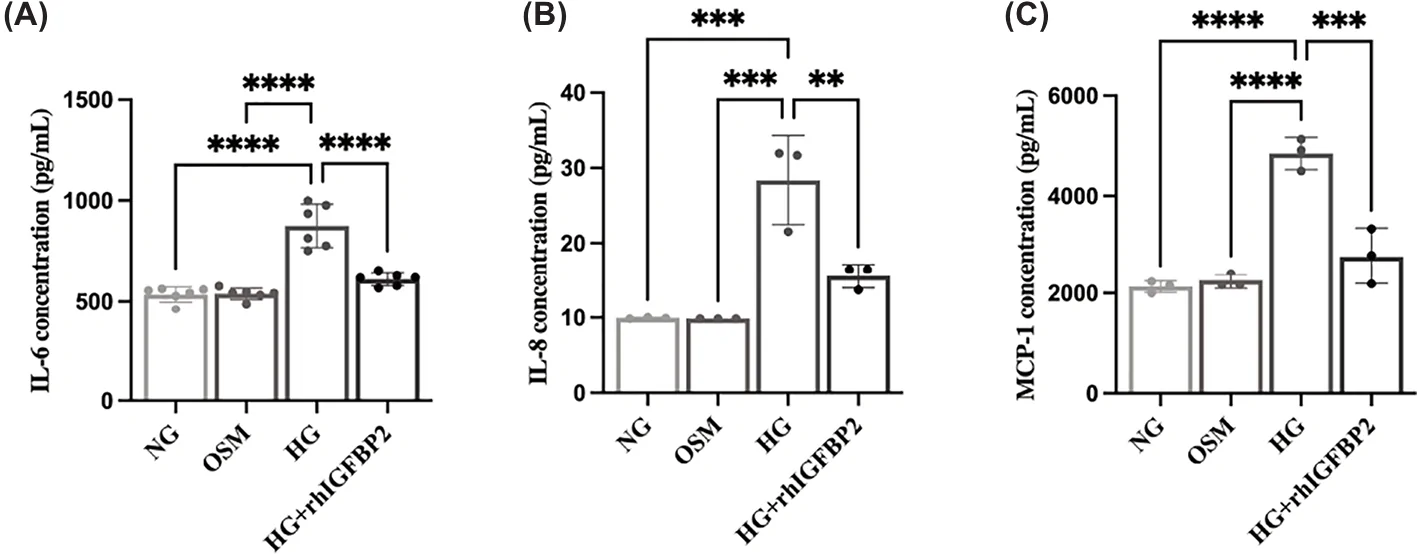
rhIGFBP2 suppresses HG-induced inflammatory cytokine secretion in HUVECs (**A**) IL-6 concentration in the cell culture supernatant. (**B**) IL-8 concentration in the cell culture supernatant. (**C**) MCP-1 concentration in the cell culture supernatant. NG, normal glucose; OSM, osmotic control; HG, high glucose; rhIGFBP2, recombinant human IGFBP2; IL-6, interleukin-6; IL-8, interleukin-8; MCP-1, monocyte chemoattractant protein-1. For IL-6, data are presented as the mean ± SD from six independent experiments. For IL-8 and MCP-1, data are presented as the mean ± SD from three independent experiments. **P<0.01, ***P<0.001, ****P<0.0001, as indicated.

### rhIGFBP2 improves cell viability and reduces HG-induced apoptosis in HUVECs

We first assessed the protective effect of rhIGFBP2 on cell viability using the CCK-8 assay. HG exposure reduced HUVEC viability, and vehicle treatment did not reverse this effect. In contrast, rhIGFBP2 treatment partially restored cell viability under HG conditions (Figure 5A). We further evaluated HUVEC apoptosis using Annexin V-FITC/PI staining followed by flow cytometry. Representative flow cytometry plots showed that HG increased the proportion of apoptotic cells compared with NG, whereas rhIGFBP2 treatment reduced HG-induced apoptosis (Figure 5B). Quantitative analysis confirmed that the percentage of total apoptotic cells was significantly increased by HG and significantly reduced after rhIGFBP2 treatment (Figure 5C). These results indicate that rhIGFBP2 protects HUVECs from HG-induced loss of viability and apoptosis.

**Figure 5:**
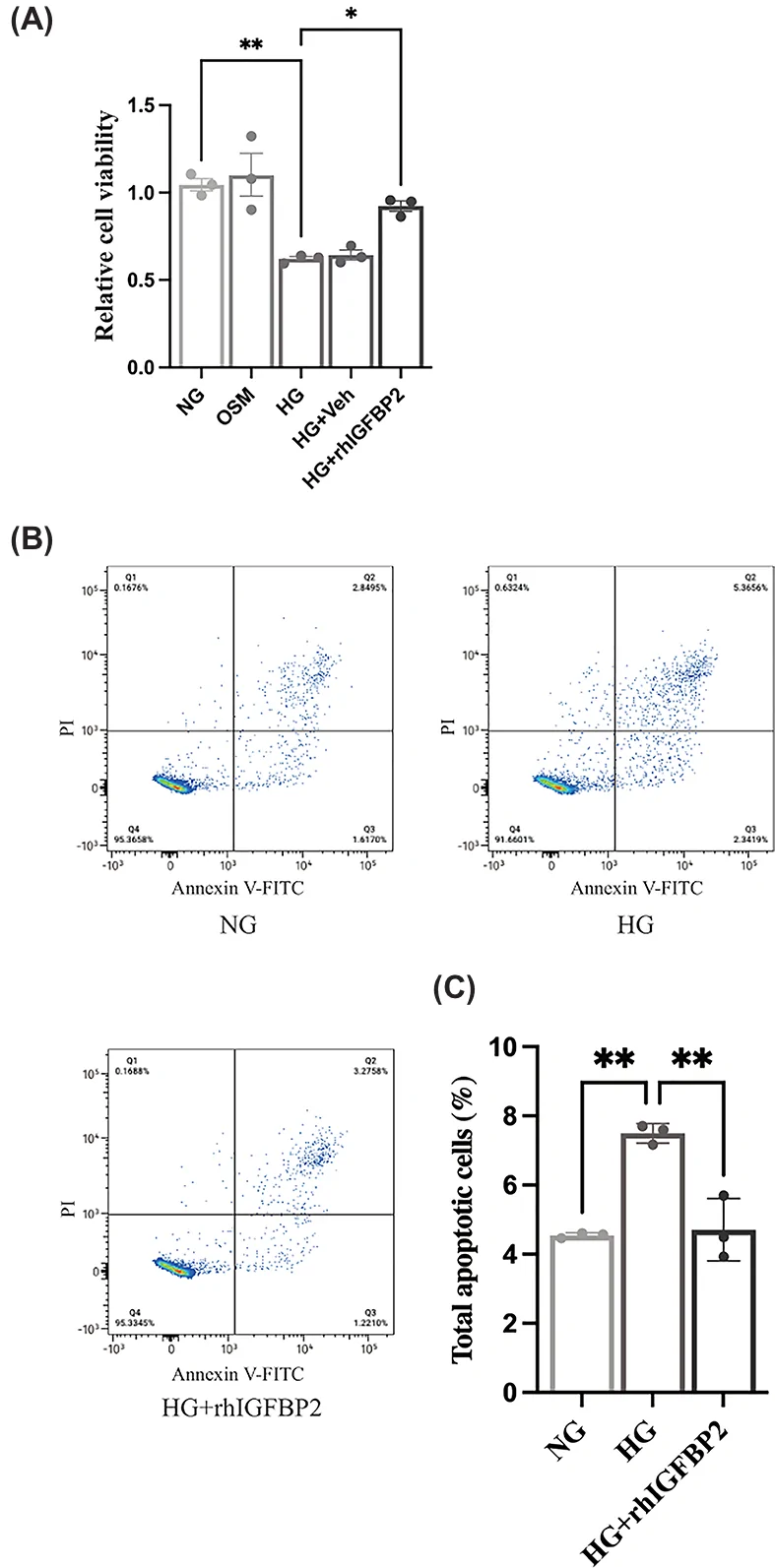
rhIGFBP2 improves cell viability and reduces HG-induced apoptosis in HUVECs (**A**) Cell viability of HUVECs assessed by the CCK-8 assay under NG, OSM, HG, HG + Veh, and HG + rhIGFBP2 conditions. (**B**) Representative flow cytometry plots of Annexin V-FITC/PI staining in NG, HG, and HG + rhIGFBP2 groups. (**C**) Quantification of the percentage of total apoptotic cells. Early apoptotic cells were defined as Annexin V-FITC-positive/PI-negative cells, and late apoptotic cells were defined as Annexin V-FITC-positive/PI-positive cells. The percentage of total apoptotic cells was calculated as the sum of early and late apoptotic cells. NG, normal glucose; OSM, osmotic control; HG, high glucose; Veh, PBS vehicle for rhIGFBP2; rhIGFBP2, recombinant human IGFBP2. Data in A and C are presented as the mean ± SD from three independent experiments. *P<0.05, **P<0.01, as indicated.

### rhIGFBP2 shows comparable effects to insulin on the Akt/eNOS/NO pathway under HG conditions

To compare the effect of rhIGFBP2 with that of insulin, a known activator of the PI3K/Akt pathway, HUVECs were treated with insulin or rhIGFBP2 after HG exposure. HG decreased the phosphorylation levels of Akt and eNOS, whereas both insulin and rhIGFBP2 restored Akt and eNOS phosphorylation under HG conditions (Figure 6A,B). Consistently, HG reduced NO production, while insulin and rhIGFBP2 both increased NO levels compared with HG alone (Figure 6C). Although insulin showed a slightly stronger tendency to restore NO production, no significant difference was observed between the insulin-treated and rhIGFBP2-treated groups. These findings suggest that rhIGFBP2 restores the Akt/eNOS/NO signaling axis under HG conditions, with an effect broadly comparable to that of insulin in this experimental setting.

**Figure 6:**
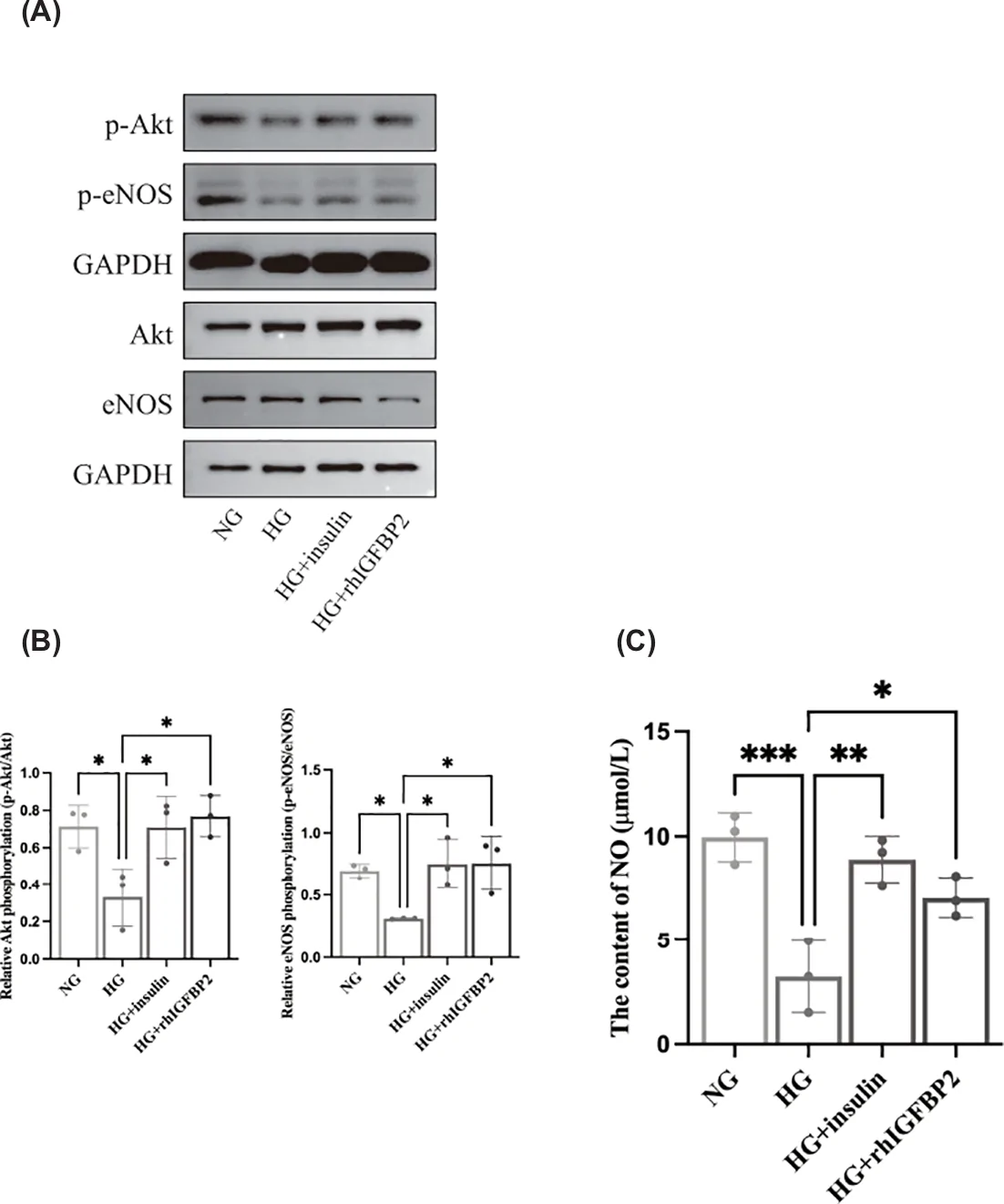
Comparison of the effects of insulin and rhIGFBP2 on the Akt/eNOS/NO pathway under HG conditions (**A**) Representative Western blot images showing p-Akt, total Akt, p-eNOS, total eNOS, and GAPDH protein levels in HUVECs under NG, HG, HG + insulin, and HG + rhIGFBP2 conditions. Insulin was used at a final concentration of 1 μg/ml, and rhIGFBP2 was used at a final concentration of 200 ng/ml. (**B**) Quantification of the p-Akt/Akt and p-eNOS/eNOS ratios. (**C**) NO production in the cell culture supernatant. NG, normal glucose; HG, high glucose; rhIGFBP2, recombinant human IGFBP2; NO, nitric oxide. Data are presented as the mean ± SD from three independent experiments. *P<0.05, **P<0.01, ***P<0.001, as indicated.

### Effect of rhIGFBP2 on HG-induced p38 MAPK phosphorylation

Given the potential relationship between PI3K/Akt signaling and p38 MAPK, we next examined p38 MAPK phosphorylation. HG significantly increased the p-p38/p38 ratio compared with NG, indicating activation of p38 MAPK under HG conditions (Figure 7A,B). rhIGFBP2 treatment showed a tendency to reduce HG-induced p38 MAPK phosphorylation; however, the difference between HG and HG + rhIGFBP2 was not statistically significant (Figure 7B). These data suggest that HG activates p38 MAPK in HUVECs, but the protective effect of rhIGFBP2 observed in the present study is more closely associated with restoration of the PI3K/Akt/eNOS pathway than with a statistically significant reversal of p38 MAPK phosphorylation.

**Figure 7:**
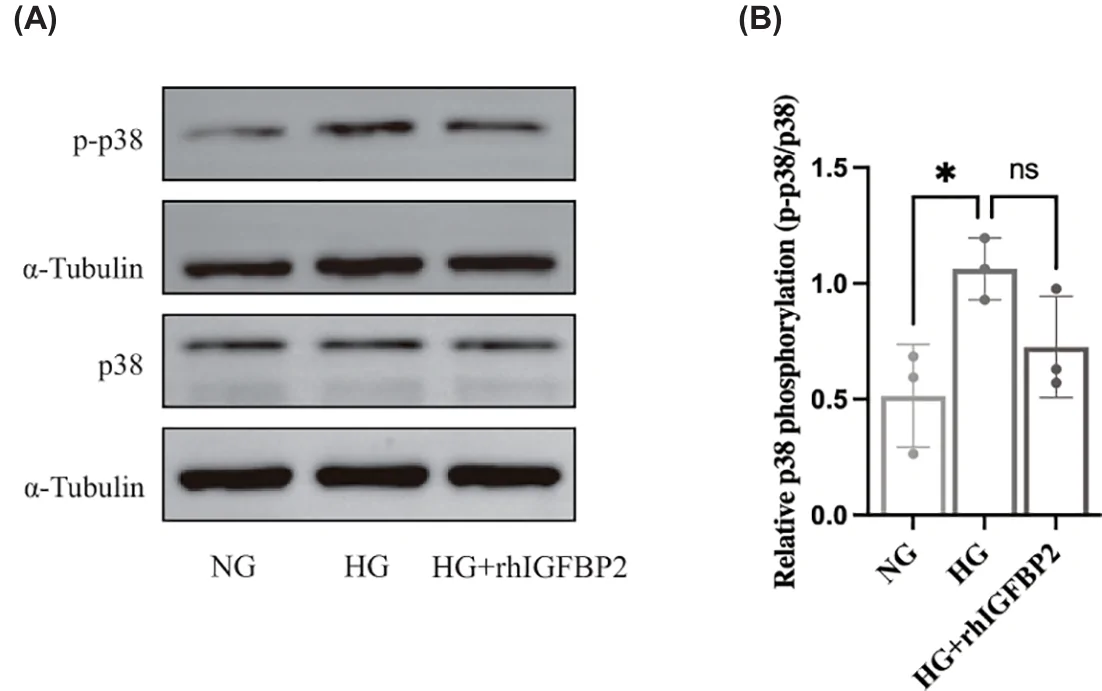
Effect of rhIGFBP2 on HG-induced p38 MAPK phosphorylation in HUVECs (**A**) Representative Western blot images showing p-p38 MAPK, total p38 MAPK, and α-Tubulin protein levels in HUVECs under NG, HG, and HG + rhIGFBP2 conditions. (**B**) Quantification of the p-p38/p38 ratio. NG, normal glucose; HG, high glucose; rhIGFBP2, recombinant human IGFBP2; MAPK, mitogen-activated protein kinase. Data are presented as the mean ± SD from three independent experiments. *P<0.05; ns, not significant.

### PI3K inhibition abolishes the protective signaling effect of rhIGFBP2

To determine whether the protective effect of rhIGFBP2 depends on PI3K/Akt signaling, we used the PI3K inhibitor LY294002. Under HG conditions, rhIGFBP2 restored Akt and eNOS phosphorylation. However, pretreatment with LY294002 markedly suppressed Akt and eNOS phosphorylation and abolished the rhIGFBP2-induced activation of this pathway (Figure 8A,B). The p-Akt/Akt and p-eNOS/eNOS ratios in the HG + rhIGFBP2 + LY294002 group were substantially lower than those in the HG + rhIGFBP2 group, indicating that PI3K inhibition blocked the signaling effect of rhIGFBP2.

**Figure 8:**
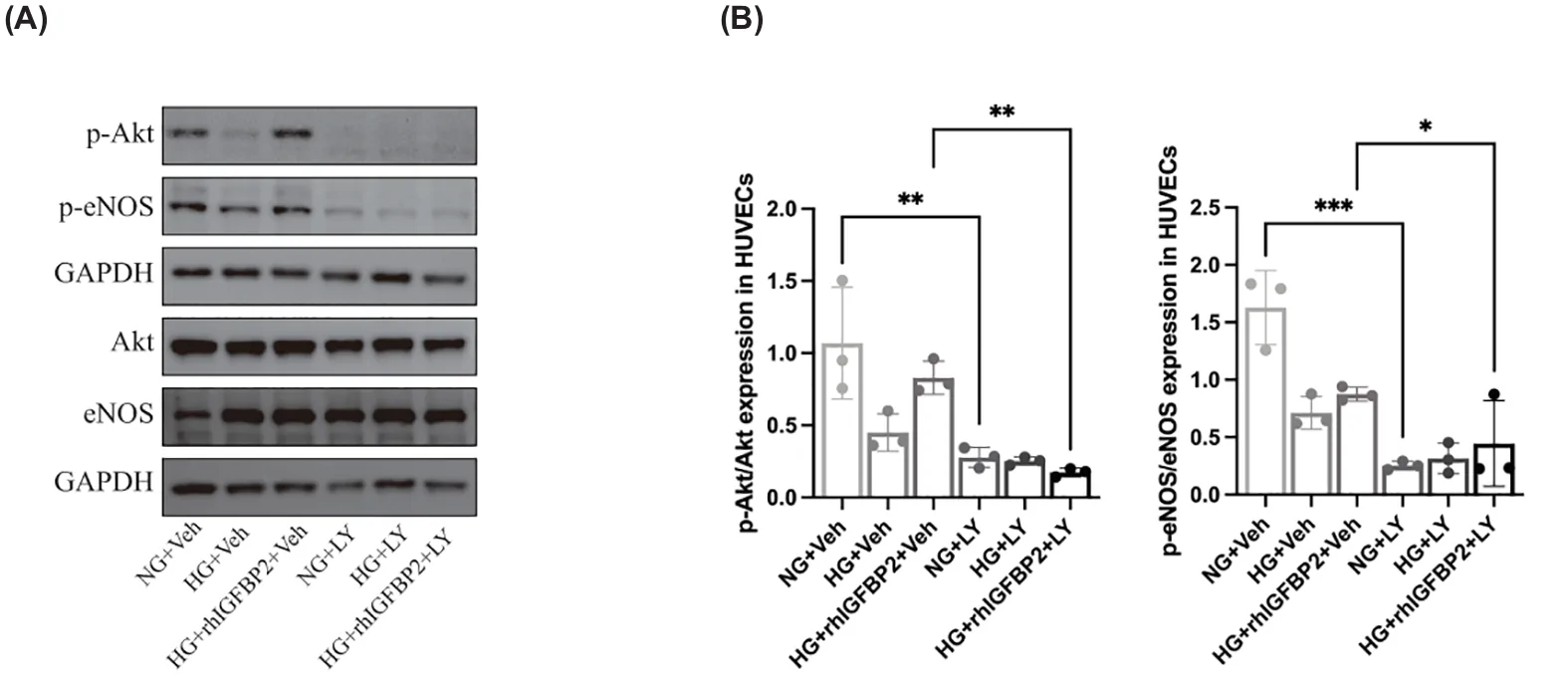
PI3K inhibition abolishes rhIGFBP2-induced activation of Akt/eNOS signaling under HG conditions (**A**) Representative Western blot images showing p-Akt, total Akt, p-eNOS, total eNOS, and GAPDH protein levels in HUVECs under NG + Veh, HG + Veh, HG + rhIGFBP2 + Veh, NG + LY, HG + LY, and HG + rhIGFBP2 + LY conditions. (**B**) Quantification of the p-Akt/Akt and p-eNOS/eNOS ratios. LY, LY294002; Veh, DMSO vehicle for LY294002; NG, normal glucose; HG, high glucose; rhIGFBP2, recombinant human IGFBP2. Data are presented as the mean ± SD from three independent experiments. *P<0.05, **P<0.01, ***P<0.001, as indicated.

Together, these results demonstrate that rhIGFBP2 protects HUVECs against HG-induced endothelial dysfunction mainly through activation of the PI3K/Akt/eNOS pathway, leading to restored NO production, reduced inflammatory activation, and decreased apoptosis. The proposed protective mechanism is summarized in Figure 9.

**Figure 9:**
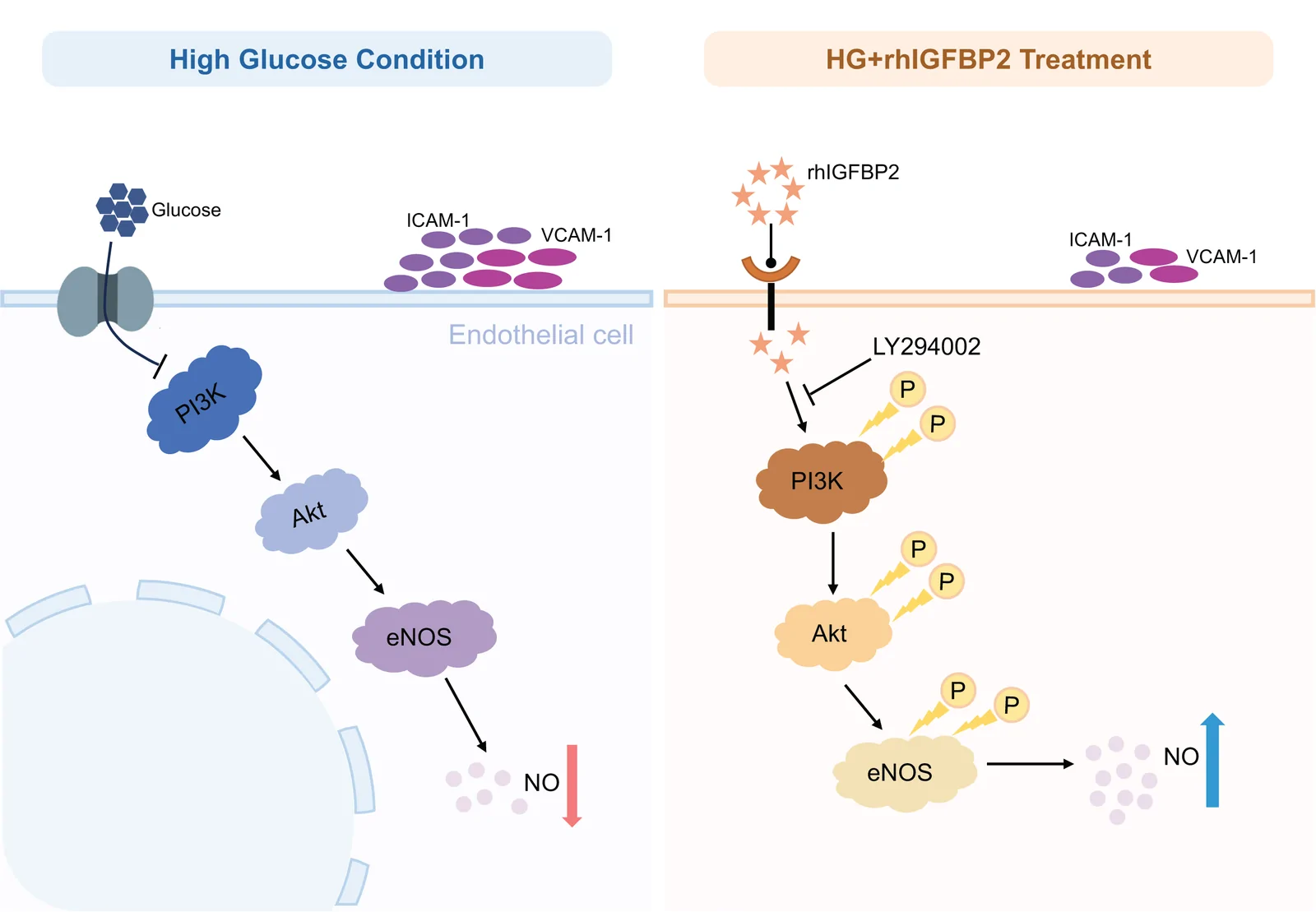
Proposed mechanism by which rhIGFBP2 protects against HG-induced endothelial dysfunction Under HG conditions, the PI3K/Akt/eNOS signaling pathway is suppressed, leading to reduced NO production and increased endothelial inflammatory activation. rhIGFBP2 treatment activates the PI3K/Akt/eNOS pathway, restores NO production, and attenuates HG-induced endothelial dysfunction. The PI3K inhibitor LY294002 blocks rhIGFBP2-induced activation of Akt/eNOS signaling, supporting the involvement of the PI3K/Akt/eNOS pathway in the protective effect of rhIGFBP2. HG, high glucose; rhIGFBP2, recombinant human IGFBP2; NO, nitric oxide.

## Discussion

The present study demonstrates that rhIGFBP2 protects HUVECs against HG-induced endothelial dysfunction mainly through activation of the PI3K/Akt/eNOS signaling pathway. HG exposure suppressed PI3K, Akt, and eNOS phosphorylation, reduced NO production, increased adhesion molecule expression, promoted inflammatory cytokine secretion, and enhanced apoptosis. rhIGFBP2 treatment reversed many of these HG-induced alterations, including restoration of Akt/eNOS phosphorylation and NO production, suppression of ICAM-1, VCAM-1, and SELE expression, reduction of IL-6, IL-8, and MCP-1 secretion, and attenuation of apoptosis. Importantly, the protective signaling effect of rhIGFBP2 was abolished by the PI3K inhibitor LY294002, supporting a central role for the PI3K/Akt/eNOS axis in rhIGFBP2-mediated endothelial protection.

Endothelial dysfunction is an early and critical event in diabetic vascular complications [31,32]. Reduced NO bioavailability, increased endothelial inflammation, enhanced leukocyte adhesion, and endothelial apoptosis collectively contribute to vascular injury under hyperglycemic conditions [10,31,32]. The PI3K/Akt/eNOS pathway is a key signaling axis for maintaining endothelial homeostasis, and Akt-mediated eNOS phosphorylation promotes NO production [13–17]. In the present study, exposure to 33 mM glucose for 24 h reduced Akt and eNOS phosphorylation, together with a decrease in NO production. rhIGFBP2 restored Akt/eNOS phosphorylation and increased NO production under HG conditions. Since NO is closely involved in vascular relaxation and also contributes to the suppression of endothelial inflammation and apoptosis [15–17], the recovery of NO production may be an important mechanism by which rhIGFBP2 attenuates HG-induced endothelial injury.

Inflammation is a key feature of HG-induced endothelial injury [10,31,32]. In the present study, HG increased the expression of the adhesion molecules ICAM-1, VCAM-1, and SELE, accompanied by enhanced monocyte adhesion to endothelial cells and increased secretion of IL-6, IL-8, and MCP-1. rhIGFBP2 attenuated these HG-induced inflammatory changes, suggesting that IGFBP2 may suppress endothelial inflammatory activation under hyperglycemic conditions. Since ICAM-1, VCAM-1, and E-selectin are involved in leukocyte recruitment and endothelial inflammatory responses, the inhibitory effect of rhIGFBP2 on these adhesion molecules may be relevant to early diabetic vascular inflammation.

Endothelial cell survival is closely related to vascular homeostasis under diabetic conditions. In the present study, HG reduced HUVEC viability and increased apoptosis, whereas rhIGFBP2 partially restored cell viability and decreased the proportion of apoptotic cells. These results suggest that rhIGFBP2 protects endothelial cells not only by improving Akt/eNOS/NO signaling but also by attenuating HG-induced apoptotic injury. Since the PI3K/Akt pathway is closely associated with cell survival signaling, activation of this pathway may contribute to the anti-apoptotic effect of rhIGFBP2 observed in HUVECs [23–26,33].

Insulin is a well-established activator of endothelial PI3K/Akt signaling and promotes eNOS phosphorylation and NO production through the insulin receptor/PI3K/Akt pathway [34]. In the present study, insulin was used as a positive comparator to evaluate the effect of rhIGFBP2 on this signaling axis under HG conditions. Both insulin and rhIGFBP2 restored Akt/eNOS phosphorylation and increased NO production after HG exposure. Although insulin showed a slightly stronger trend in some readouts, the difference between insulin and rhIGFBP2 was not statistically significant. These findings suggest that rhIGFBP2 can activate the Akt/eNOS/NO pathway under hyperglycemic conditions with an effect broadly comparable to insulin in this *in vitro* model. Rather than replacing insulin, IGFBP2 may act as an additional regulator of endothelial PI3K/Akt/eNOS signaling in the context of diabetic vascular dysfunction.

We also examined p38 MAPK phosphorylation because HG has been reported to activate p38 MAPK in human endothelial cells and because p38 MAPK is involved in cellular stress responses under hyperglycemic conditions [35]. In our study, HG significantly increased p38 MAPK phosphorylation, indicating activation of stress-related signaling in HUVECs. rhIGFBP2 treatment showed a tendency to reduce HG-induced p38 phosphorylation, but the difference between the HG and HG + rhIGFBP2 groups was not statistically significant. Therefore, our data suggest that p38 MAPK activation is involved in the HG-induced stress response, but the protective effect of rhIGFBP2 in this model appears to be more closely associated with restoration of PI3K/Akt/eNOS signaling than with significant inhibition of p38 MAPK phosphorylation. Further studies are needed to determine whether p38 MAPK interacts with the IGFBP2-PI3K/Akt/eNOS axis under different treatment durations or injury intensities.

The upstream mechanism by which IGFBP2 activates PI3K/Akt signaling in endothelial cells remains an important question. IGFBP2 was initially recognized as a regulator of IGF bioavailability, but increasing evidence indicates that IGFBP2 also has IGF-independent functions [23–26,36]. A previous study demonstrated that IGFBP2 contains an Arg-Gly-Asp (RGD) motif and can interact with α5β1-integrin on the cell surface in an IGF-independent manner [37]. Another study further showed that the interaction between IGFBP2 and integrin α5 is essential for IGFBP2-induced cell mobility and that mutation of the RGD motif disrupts this interaction [38]. In addition, IGFBP2 has been linked to FAK-related signaling in non-small cell lung cancer cells [39], and broader reviews of IGFBP biology support the concept that IGFBPs can regulate intracellular signaling through both IGF-dependent and IGF-independent mechanisms [23–26,36]. Based on these findings, it is possible that rhIGFBP2 activates Akt/eNOS signaling in endothelial cells through integrin-associated signaling, IGF-related mechanisms, or both. However, the present study did not directly identify the receptor or membrane-associated binding partner responsible for rhIGFBP2 action in HUVECs. Future studies using integrin-blocking antibodies, IGF-1R inhibition, receptor knockdown, or RGD-mutant IGFBP2 will be necessary to define the upstream receptor mechanism.

The present findings may have potential clinical implications for diabetic vascular complications. Hyperglycemia-induced endothelial dysfunction contributes to the initiation and progression of diabetic macrovascular and microvascular disease [31,32]. By restoring PI3K/Akt/eNOS signaling, improving NO production, reducing endothelial inflammation, and attenuating apoptosis, IGFBP2 may represent a potential endogenous protective factor against diabetic vascular injury. Previous clinical and experimental studies have linked IGFBP2 to metabolic regulation and cardiovascular or cardiorenal complications in diabetes-related contexts [24,29,30]. In the future, circulating IGFBP2 levels may also be explored as a biomarker reflecting vascular protection or endothelial stress in patients with diabetes. Moreover, therapeutic strategies aimed at enhancing IGFBP2 signaling or mimicking its endothelial protective effect may provide a new direction for preventing hyperglycemia-associated vascular injury. Nevertheless, these possibilities remain speculative and require validation in animal models and clinical studies.

The present study has several limitations. First, all experiments were performed in HUVECs, which may not fully represent endothelial cells from arterial, microvascular, or tissue-specific vascular beds affected by diabetes. Second, the study used an *in vitro* HG stimulation model, which cannot fully reproduce the complex metabolic, inflammatory, hemodynamic, and cellular interactions present in diabetic vascular disease *in vivo*. Third, although the key experiments were independently repeated three times, the relatively small number of biological replicates is a limitation and should be considered when interpreting the findings. Finally, the upstream receptor responsible for rhIGFBP2-induced PI3K/Akt/eNOS activation was not identified, and the potential interaction between IGFBP2 signaling and p38 MAPK requires further investigation.

In conclusion, the present study demonstrates that rhIGFBP2 attenuates HG-induced endothelial dysfunction by activating the PI3K/Akt/eNOS pathway. rhIGFBP2 restored NO production, suppressed adhesion molecule expression and inflammatory cytokine secretion, and reduced apoptosis in HUVECs exposed to HG. These findings identify IGFBP2 as a potential mediator of endothelial protection under diabetic conditions and provide a basis for future studies exploring IGFBP2-related signaling as a therapeutic target for diabetic vascular complications.

## Data Availability

The datasets used and/or analyzed during the current study are available from the corresponding author on reasonable request.

## Abbreviations

CCK-8: Cell Counting Kit-8
eNOS: endothelial nitric oxide synthase
HG: high glucose
HUVECs: human umbilical vein endothelial cells
ICAM-1: intercellular adhesion molecule-1
IGFBP2: insulin-like growth factor-binding protein 2
IL-6: interleukin-6
IL-8: interleukin-8
MCP-1: monocyte chemoattractant protein-1
NG: normal glucose
NO: nitric oxide
OSM: osmotic control
RGD: arginine-glycine-aspartic acid
rhlGFBP2: recombinant human insulin-like growth factor-binding protein 2
ROS: reactive oxygen species
SD: standard deviation
PI3K: phosphoinositide 3-kinase
VCAM-1: vascular cell adhesion molecule-1
